# Perceived and objective neighborhood support for outside of school physical activity in South African children

**DOI:** 10.1186/s12889-016-2860-0

**Published:** 2016-06-01

**Authors:** Monika Uys, Stephanie T. Broyles, Catherine E. Draper, Sharief Hendricks, Dale Rae, Nirmala Naidoo, Peter T. Katzmarzyk, Estelle V. Lambert

**Affiliations:** Division of Exercise Science and Sports Medicine, Department of Human Biology, Faculty of Health Sciences, University of Cape Town, Boundary Road, Newlands, 7700 South Africa; Pennington Biomedical Research Center, 6400 Perkins Road, Baton Rouge, 70808 LA USA; Division of Physiotherapy, Department of Health and Rehabilitation Sciences, University of Cape Town, F45 Old Main Building, Groote Schuur Hospital, Observatory, 7725 South Africa

## Abstract

**Background:**

The neighborhood environment has the potential to influence children’s participation in physical activity. However, children’s outdoor play is controlled by parents to a great extent. This study aimed to investigate whether parents' perceptions of the neighborhood environment and the objectively measured neighborhood environment were associated with children's moderate-to-vigorous intensity physical activity (MVPA) outside of school hours; and to determine if these perceptions and objective measures of the neighborhood environment differ between high and low socio-economic status (SES) groups.

**Methods:**

In total, 258 parents of 9–11 year-old children, recruited from the South African sample of the International Study of Childhood Obesity, Lifestyle and the Environment (ISCOLE), completed a questionnaire concerning the family and neighborhood environment. Objective measures of the environment were also obtained using Geographic Information Systems (GIS). Children wore an Actigraph (GT3X+) accelerometer for 7 days to measure levels of MVPA. Multilevel regression models were used to determine the association between the neighborhood environment and MVPA out of school hours.

**Results:**

Parents’ perceptions of the neighborhood physical activity facilities were positively associated with children’s MVPA before school (*β* = 1.50 ± 0.51, *p* = 0.003). Objective measures of neighborhood safety and traffic risk were associated with children’s after-school MVPA (*β* = −2.72 ± 1.35, *p* = 0.044 and *β* = −2.63 ± 1.26, *p* = 0.038, respectively). These associations were significant in the low SES group (*β* = −3.38 ± 1.65, *p* = 0.040 and *β* = −3.76 ± 1.61, *p* = 0.020, respectively), but unrelated to MVPA in the high SES group.

**Conclusions:**

This study found that several of the objective measures of the neighborhood environment were significantly associated with children’s outside-of-school MVPA, while most of the parents’ perceptions of the neighborhood environment were unrelated.

## Background

It is recommended that children participate in at least 60 min of moderate-to-vigorous intensity physical activity (MVPA) per day [37]. The 2008 South African National Youth Risk Behavior Survey found that only 29.3 % of adolescents participated in sufficient moderate physical activity and 43.2 % in sufficient vigorous physical activity to be beneficial to their health [25].

The social ecological model posits that physical activity behavior is determined both by individual factors as well as the social (e.g. family) and the built environment (e.g. neighborhood) [30]. While the social and built environments of neighborhoods have the potential to influence children’s participation in physical activity [12, 21, 33], children’s outdoor time has been shown to be controlled by parents to a great extent [34, 36]. For this reason, neighborhood characteristics, as well as parents’ perceptions of these characteristics, may have an impact on children’s level of physical activity [9, 34].

Characteristics of the neighborhood built environment that may be associated with physical activity include accessibility and distance to recreational facilities, opportunities to be physically active, as well as aesthetic qualities [28]. However, the distribution of and access to these physical activity-promoting facilities (for example, parks and playgrounds) are not always equal between areas of different socio-economic status (SES), and as such access to these facilities becomes an environmental justice issue [22].

The neighborhood social environment characteristics that may be associated with physical activity include the perception of social disorder (a measure of neighborhood safety including personal safety from crime and traffic) in their neighborhood [2]. The perception of high social disorder in a neighborhood may cause people to spend less time outdoors [2]. Datar et al. [8] showed that children whose parents perceived their neighborhoods as unsafe watched more television and participated in less physical activity [8]. Similarly, O'connor et al. [24] found a positive association between parental perceptions of perceived traffic safety and structure for promoting child physical activity [24].

There are limited data available on parent perceptions of the neighborhood environment and children’s physical activity in countries with low-income settings. The aims of this study were to (1) assess whether parents’ perceptions of the neighborhood environment were associated with children’s out-of-school hours and weekend day MVPA, (2) assess whether objective measures of the neighborhood environment were associated with children’s out-of-school hours and weekend day MVPA, and (3) examine whether these associations differ between different income settings.

## Methods

### Context

The analyses presented here are based on data that were collected in Cape Town for the South African site of the International Study of Childhood Obesity, Lifestyle and the Environment (ISCOLE). ISCOLE was designed to determine relationships between lifestyle behaviors and obesity in a multi-country study of 9–11 year-old children, and to investigate the influence of characteristics such as behavioral settings, and physical, social and policy environments, on the observed relationships within and between countries. Data were collected at sites from 12 countries (~500 children per site) from five major regions of the world (Eurasia, Africa, Europe, Latin America, North America, and the Pacific) [17].

The project was presented to the Western Cape Education Department (WCED) for approval. Thereafter, schools were randomly selected within five SES strata. Schools are classified into quintiles by the WCED according to the SES of the surrounding neighborhood, with quintile one representing the lowest SES and quintile five the highest. At least four schools were randomly selected from each stratum for a total of 20 schools. Children in Grade 4 and/or Grade 5 who were aged between nine and 11 years were invited to participate in the study.

Data were collected from April 2012 to May 2013, incorporating all four seasons. The study was approved by the Human Research Ethics Committee of the Health Sciences Faculty of the University of Cape Town (HREC REF: 288/2011). The principals provided approval for the study to be conducted at their school, and parents or guardians provided written informed consent for their children.

### Participants

A total of 550 children (327 girls, 223 boys) from 20 schools, aged 9–11 years old, participated in the South African arm of ISCOLE, of which 258 children (145 girls, 113 boys) remained in the analytical dataset after excluding participants without valid accelerometry (*n* = 34), a valid home address reported by parents on the questionnaire (*n* = 187) and annual family income reported by parents (*n* = 71). The mean age was 10.2 (0.6) years. There are two reasons for the relatively high number of participants excluded due to the lack of a valid home address. The first is due to missing or incomplete data (questionnaires not fully completed by parents) (*n* = 76) and the second reason is the inability to geocode certain addresses, especially for children living in informal settlements (*n* = 111). However, the children in the analytical sample were not different from the remainder of the sample, except for height and MVPA (*p* < 0.05). The proportion of families from low-income households and higher quintile schools were over-represented within the analytical sample compared to the rest of the sample (*P* < 0.01).

### Demographic information

Parents or guardians completed a demographic and family health questionnaire developed for ISCOLE [17], which included information on basic demographics, ethnicity, family health and socio-economic factors. For this paper, we report on age, self-reported parental body mass index (BMI), parental education and parental employment status. SES was based on annual household income and was reported by the parent or guardian using a monetary scale in the currency of each country. Each country-specific income scale was collapsed into four levels to facilitate multi-country analysis [18]. The four levels were: < ZAR11500 (≈ < US$ 970.31; level 1), ZAR11500 – ZAR30000 (≈ US$ 970.31–2531.25; level 2), ZAR30001 – ZAR300000 (≈ US$ 2531.33–US$ 25312.50; level 3) and > ZAR300000 (≈ > US$ 25312.50; level 4). The top two and bottom two levels were combined to derive low and high SES categories for analysis.

### Physical activity measurements

#### Objective physical activity measurement

Objective physical activity measurements were obtained using accelerometers (Actigraph GT3X+, Pensacola, Florida, USA). Children were asked to wear accelerometers for seven consecutive days (plus an initial familiarization day and the morning of the final day), including two weekend days. Accelerometers were attached to flexible belts and worn around the waist on the right hip at all times (including during sleep), except during bathing and other aquatic activities. After removal of sleep time using a validated algorithm [1], valid wear time was defined as at least four days with a minimum of 10 h of awake wear time per day, including at least one weekend day. Data were processed using 15 s epochs. Physical activity intensity cut-points were applied to the data to determine the amount of time spent in MVPA. The MVPA cut-point was ≥ 574 counts per 15 s epoch [14]. Time spent in MVPA was calculated for before school and after-school periods on weekdays specific for each participant and school. Before school is considered from wake time until school start time, after-school is considered from school end time until bed time and weekend is a combination of Saturday and Sunday wake time until bed time.

#### Mode of transport to school

Participants completed a diet and lifestyle questionnaire that included questions related to physical activity, sedentary behavior, food consumption, sleep, health and well-being [17]. Children were asked questions about their journey to school, including their mode of transport for the main part of the journey to school (‘walking’, ‘bicycle, roller-blade, skateboard or scooter’, ‘bus, train, tram, underground or boat’, ‘car, motorcycle or scooter’ or ‘other’) as well as how long it took them to travel to school (‘<5 min’, ‘5-15 min’, ‘16–30 min’, 31 min–1 h’, ‘>1 h’). Modes of transport to school were grouped into active transport combining ‘walking’ and ‘bicycle, roller-blade, skateboard or scooter’; and motorized transport which comprised of ‘bus, train, tram, underground or boat’ and ‘car, motorcycle or scooter’. None of the participants selected the ‘other’ option.

### Perceived neighborhood and home environments

Parents or guardians completed a neighborhood and home environment questionnaire, which was adapted from the Neighborhood Impact on Kids (NIK) study survey [27] which drew on questions from other validated instruments [26, 29, 32]. The questionnaire included items related to neighborhood social capital, the home social environment, the home and neighborhood food environments, the home and neighborhood physical activity environment, and neighborhood built environment [17]. For this study, we used information on parents’ perception about the neighborhood environments relating to physical activity, as shown in Table 1. The following were derived from the neighborhood and home environment questionnaire:

**Table 1:** Questionnaire items used to construct parent perceptions

Perceptions	Scale^a^	Items
Proximity to community facilities	1–5 min, 6–10 min, 11–20 min, 21–30 min, >30 min, and don’t know	Parents estimated the length of time it took to walk from home to the nearest sporting venues, recreational facilities and parks by selecting one of six options
Neighborhood safety	Four-point scale ranging from strongly disagree = 0 to strongly agree = 3	1. ‘There is a high crime rate’ 2. ‘Streets have good lighting at night’ 3. ‘I’m afraid of my child being taken or hurt by a stranger on local streets’ 4. ‘I’m afraid of my child being taken or hurt by a stranger in my yard, driveway, or common area’ 5. ‘I’m afraid of my child being taken or hurt by a stranger in a local park’ 6. ‘I’m afraid of my child being taken or hurt by a known “bad” person (adult or child) in my neighborhood’ The one positive question (#2) was reverse coded so that a high score for neighborhood safety indicated a perceived unsafe neighborhood.
Traffic safety	Four-point scale ranging from strongly disagree = 0 to strongly agree = 3	1. ‘The speed of traffic on most streets is usually slow (50 kph or less)’ 2. ‘Most drivers go faster than the posted speed limits’ 3. ‘The traffic makes it difficult or unpleasant for my child to walk’ 4. ‘There are crosswalks and robots (traffic lights) on busy streets’ Negatively phrased questions (#2 and 3) were reverse coded so that a high traffic safety score indicated that the neighborhood’s roads were perceived as safe.
Walkability	Four-point scale ranging from strongly disagree = 0 to strongly agree = 3	1. ‘There are shops, stores, markets and places to buy things I need within easy walking distance of my home/house’ 2. ‘There is a bus, taxi, or train stop within walking distance from my home’ 3. ‘There are sidewalks on most streets’ 4. ‘There are many different routes for getting from place to place’ 5. ‘There are many interesting things to look at while walking in my neighborhood’ 6. ‘There are many places to go within easy walking distance from my home’
Social cohesion	Section 1: Five-point scale ranging from strongly disagree = 0 to strongly agree = 4 Section 2: Four-point scale ranging from not at all = 1 to extremely well = 4 for item 1; seven-point scale from never = 0 to almost every day = 7 for item 2, which were collapsed into a five-point scale to be consistent with the other items Section 3: Five-point scale ranging from very unlikely = 0 to very likely = 4	Section 1: 1. ‘People around my neighborhood are willing to help their neighbors’ 2. ‘This is a close-knit neighborhood’ 3. ‘People in my neighborhood can be trusted’ 4. ‘People in my neighborhood generally don’t get along with each other’ 5. ‘People in my neighborhood do not share the same values, attitudes or beliefs’. Negatively phrased questions (#4 and 5) were reverse coded Section 2: 1. ‘In general, how well do you feel you know your neighbors?’ 2. ‘About how often do you talk to or visit your immediate neighbors (people in the 10–20 households that live closest to you)?’ Section 3: 1. ‘If a group of neighborhood children were skipping school and hanging out on a street corner, how likely is it that your neighbors would do something about it?’ 2. ‘If some children were spray-painting graffiti on a local building, how likely is it that your neighbors would do something about it?’ 3. ‘If a child was showing disrespect to an adult, how likely is it that people in your neighborhood would scold that child?’ 4. ‘If there was a fight in front of your house and someone was being beaten or threaten, how likely is it that your neighbors would break it up?’ 5. ‘Suppose that because of budget cuts the fire station closest to you home was going to be closed down by the city. How likely is it that neighborhood residents would organize to try to do something to keep the fire station open?’
Family support for physical activity	Items used individually, not combined into a scale. Never, 1–2 days, 3–4 days, 5–6 days, every day	‘How often do you or another adult in the household: 1. watch your child participate in physical activity or sports; 2. encourage your child to do sports or physical activity; 3. provide transport to a place where your child can do physical activity or 4. play sports and do a physical activity or play sports with your child’.

^a^Scales were derived from [26, 27, 29, 32]

### Objectively measured neighborhood environment

#### Facilities for physical activity

Geographic Information Systems (GIS) (ArcGIS version 10.1) [13] were used to identify the presence of facilities for physical activity (sporting venues, recreational facilities and parks) within a residential buffer. The source of the point data was the City of Cape Town. A 500 m radial buffer was created around each participant’s home address as this distance is between one-third and one-quarter mile, a distance that provides easy access (~10 min of travel time) for children travelling on foot or bike [11, 38].

#### Neighborhood safety

Crime statistics for the 2012/2013 period for the neighborhood in which each address is located were obtained from www.crimestatssa.com, which provides annual crime statistics released by the South African Police Service (SAPS). The sample represented nineteen neighborhoods. The number of children per neighborhood ranged from two to 45. The crime statistics used were the annual number of all crimes (including contact and contact-related crime, property-related crime, crime detected as result of police action and other serious crimes) broken down by neighborhood.

#### Traffic risk

The numbers of motor vehicle accidents for the neighborhood (by police precinct) in which each address was located during the study period (April 2012–May 2013) were obtained from the Transport for Cape Town Division of the City of Cape Town. The sample represented nineteen police precincts and the number of children per police precinct ranged from two to 47.

### Data analyses

The children of the sub-group comprised of parents who completed the questionnaires, reported on neighborhood perceptions, annual family income and provided a valid home address were compared to the remainder of the sample, using independent t-tests for body composition and objectively measured physical activity levels. The families were compared for income levels and school quintiles using Chi Square analysis.

Descriptive statistics were computed for children’s physical activity data. Multilevel linear regression models were used to determine the association between parents’ perceptions of the neighborhood environment and accelerometry-based MVPA minutes at three different time points: before school, during after-school hours and weekend days. Schools and participants were included as levels in the models. Models were adjusted for age, gender, SES (as measured by family income). Similar models were used to determine associations between the objective neighborhood environment and MVPA before school, during after-school hours and weekend days. To test the interaction between neighborhood constructs (perceived and objective) and SES, the cross-product term of both variables was included in the models. When a significant interaction was found, separate models were fitted for high and low SES. Multilevel logistic regression models were used to determine the association between objective measures of neighborhood safety and traffic and mode of transport to school (active versus motorized transport). These models included the same levels (schools and participants) as the linear models, and were also adjusted for age, gender, SES (as measured by family income). Inter-item reliability for the parental perception scales was assessed with Cronbach’s alpha. T-tests were done to determine if there were differences in minutes of MVPA, mode of transport to school and objective measures of the neighborhood environment between the high and low SES groups. All analyses were performed using Stata (v.12, StataCorp, Texas, USA). Data reported as beta coefficient and standard error. Results were considered significant at *p* < 0.05.

## Results

### Parent characteristics

Data were collected from 258 parents. The mean age of mothers was 37.8 ± 5.9 years and 41.0 ± 6.2 years for fathers. The mean BMIs for mothers and fathers were 28.3 ± 7.0 and 28.8 ± 5.0 kg/m^2^, respectively. A total of 47 % of mothers and 66 % of fathers were employed full-time, respectively. The majority of families (38 %) had more than two children at home, and 67 % of parents were currently married.

### Children’s physical activity

#### Objective physical activity measurement

On average, participants engaged in 6 ± 4 min of MVPA before school and 38 ± 18 min after-school so that their total out-of-school MVPA on week days was 45 ± 20 min. On weekend days, participants accumulated 61 ± 32 min of MVPA. The participants’ average daily minutes of out-of-school and weekend MVPA taking into account SES are presented in Table 2.

**Table 2:** Moderate-to-vigorous intensity physical activity of low and high SES participants before and after school and on weekends

Time-point	Average daily minutes of MVPA			
	Low SES (*n* = 176)	High SES (*n* = 82)	*P-*value	Overall (*n* = 258)
Before school (min/day)	6 ± 5	6 ± 4	0.898	6 ± 4
After school (min/day)	39 ± 19	36 ± 16	0.152	38 ± 18
Weekend days (min/day)	62 ± 33	57 ± 29	0.191	61 ± 32

Data are presented as mean ± SD

*MVPA* moderate- to vigorous-intensity physical activity, *SES* socio-economic status

#### Mode of transport to school

Overall, approximately half (47 %) of the children use active transport to get to school. Active transport to school is much more prominent in children from the low SES group (58 %), while only 24 % of children in the high SES group actively travel to school, as seen in Table 3. There were no significant associations between mode of transport to school and objective measures of neighborhood safety and traffic.

**Table 3:** Mode of transport to school

Mode of transport to school	Low SES (*n* = 176)	High SES (*n* = 82)	*P-*value	Overall (*n* = 258)
Active transport	58	24	<0.0001	47
Motorized transport	42	76	<0.0001	53

Data reported as percentages. Active transport = walking, bicycle, roller-blade, skateboard or scooter; motorized transport = bus, train, tram, car, motorcycle or scooter

### Parents’ perceptions of the neighborhood environment and children’s MVPA

The internal consistency of the scales created for parental constructs of the environment was generally high, with the exception of traffic safety. The Cronbach’s alpha was 0.73 for perceived proximity of community facilities, 0.79 for neighborhood safety, 0.63 for walkability and 0.83 for social cohesion. For traffic safety, the Cronbach’s alpha was 0.23. The data presented in Table 4 indicate that there were no associations between parental perceptions of the neighborhood environment and children’s MVPA except for the perception of the availability of neighborhood physical activity-related facilities and before school MVPA (*p* = 0.003). In addition, we found a significant interaction with SES at this time-point (*p* = 0.005) with a significant, positive association between before school MVPA and the number of neighborhood perceived physical activity facilities in the low SES group (*β* = 1.47, *p* = 0.005). In contrast, this association in the high SES group was not significant (*β* = −0.19, *p* = 0.554).

**Table 4:** Associations between parental perceptions of the neighborhood environment and children’s moderate- to vigorous-intensity physical activity before and after school, and on the weekend

Perceptions	MVPA								
	Before			After			Weekend		
	β (SE)^a^	*P-*value for main effect	*P-*value for SES interaction	β (SE)^a^	*P-*value for main effect	*P-*value for SES interaction	β (SE)^a^	*P-*value for main effect	*P-*value for SES interaction
Unsafe neighborhood	−0.7 (0.10)	0.485	0.703	−0.37 (0.42)	0.383	0.649	−0.73 (0.76)	0.339	0.907
Traffic safety	0.14 (0.17)	0.430	0.511	−0.32 (0.70)	0.644	0.599	−0.81 (1.28)	0.526	0.807
Walkability	0.13 (0.11)	0.241	0.184	0.70 (0.43)	0.105	0.138	0.61 (0.79)	0.438	0.358
Social cohesion	−0.05 (0.04)	0.205	0.954	−0.01 (0.17)	0.955	0.927	−0.06 (0.31)	0.853	0.795
Availability of PA facilities^b^	1.50 (0.51)	**0.003**	**0.005**	0.96 (2.26)	0.672	0.926	−1.57 (4.18)	0.706	0.244

Data reported as beta coefficient and SE

*PA* physical activity, *MVPA* moderate- to vigorous-intensity physical activity, *SES* socio-economic status

^a^Associations between each independent variable and the dependent variable, adjusted for child age and sex, SES, and clustering by school

^b^Number of PA facilities within a ~10 min walk from home. Values in bold indicate significance (p < 0.05)

### Objective measures of the neighborhood environment and children’s MVPA

Table 5 reports descriptive data of the objective measures of the neighborhood environment, by SES group. There were no significant differences in neighborhood safety, traffic safety or the availability of physical activity related facilities between the high and low SES groups.

**Table 5:** Objective measures of the neighborhood environment between high and low SES groups

Objective measurements	Low SES (*n* = 176)	High SES (*n* = 82)	*P-value*	Overall (*n* = 258)
Unsafe neighborhood (crime rates)	6095.38 ± 4026.05	6431.01 ± 4198.10	0.539	6202.05 ± 4076.33
Traffic risk (motor vehicle accidents)	103.11 ± 78.19	91.87 ± 60.72	0.251	99.53 ± 73.16
Availability of PA facilities^a^	11.57 ± 7.46	10.72 ± 8.93	0.426	11.30 ± 7.95

Data are presented as mean ± SD

^a^The actual number of PA facilities within 500 m from home objectively measured using Geographic Information Systems

Table 6 shows that none of the objective measurements of the neighborhood environment, including an unsafe neighborhood, lack of traffic safety and the availability of physical activity related facilities were associated with children’s MVPA before school (*p* = 0.121, *p* = 0.288 and *p* = 0.267, respectively). After-school MVPA, however, was significantly inversely associated with an unsafe neighborhood (*p* = 0.044) and traffic risk (*p* = 0.038), but not with the presence of PA facilities (*p* = 0.893). In addition, unsafe neighborhoods and traffic risk both had a significant SES interaction. Children in the low SES group were less active in unsafe neighborhoods (*β* = −3.38, p = 0.040) and areas with high traffic risk (*β* = −3.76, *p* = 0.020), while no such relationship was found in the high SES group (*β* = 2.00, *p* = 0.112 and *β* = 1.49, *p* = 0.227, respectively). MVPA during weekend days was not associated with any of the objective neighborhood measures (*p* = 0.315, *p* = 0.404 and *p* = 0.248, respectively).

**Table 6:** Relationship between objective measurements of the neighborhood environment and children’s moderate- to vigorous-intensity physical activity before and after school, and on the weekends

Objective measurements	MVPA								
	Before			After			Weekend		
	β (SE)^a^	*P-*value for main effect	*P-*value for SES interaction	β (SE)^a^	*P-*value for main effect	*P-*value for SES interaction	β (SE)^a^	*P-*value for main effect	*P-*value for SES interaction
Unsafe neighborhood (crime rates)	−0.53 (0.34)	0.121	0.582	−2.72 (1.35)	**0.044**	**0.021**	−2.44 (2.43)	0.315	0.661
Traffic risk (motor vehicle accidents)	−0.35 (0.33)	0.288	0.379	−2.63 (1.26)	**0.038**	**0.048**	−1.89 (2.26)	0.404	0.831
Availability of PA facilities^b^	−0.06 (0.05)	0.267	0.935	−0.03 (0.21)	0.893	0.288	−0.44 (0.38)	0.248	0.090

Data reported as beta coefficient and SE

*PA* physical activity, *MVPA* moderate- to vigorous-intensity physical activity, *SES* socio-economic status

^a^Associations between each independent variable and the dependent variable, adjusted for child age and sex, SES, and clustering by school

^b^The actual number of PA facilities within 500 m from home objectively measured using Geographic Information Systems. Values in bold indicate significance (p < 0.05)

### Family support for physical activity

Table 7 shows the results of the multilevel modelling analysis to determine whether or not there were any associations between family support and the children’s MVPA outside of school hours. None of the measurements of family support were associated with MVPA before school on weekdays or on weekend days. Providing transport to a place where children can do PA or play sports was, however, significantly associated with children’s after-school MVPA (*p* = 0.026), independent of SES.

**Table 7:** Relationship between family support for physical activity and children’s moderate- to vigorous-intensity physical activity before and after school, and on the weekends

Family support	MVPA								
	Before			After			Weekend		
	β (SE)^a^	*P-*value for main effect	*P-*value for SES interaction	β (SE)^a^	*P-*value for main effect	*P-*value for SES interaction	β (SE)^a^	*P-*value for main effect	*P-*value for SES interaction
Watch your child participate in PA or sports	0.06 (0.29)	0.837	0.273	0.89 (1.15)	0.440	0.512	0.22 (2.08)	0.915	0.111
Encourage your child to do sports or PA	0.25 (0.25)	0.316	0.204	1.01 (0.99)	0.307	0.180	−0.41 (1.82)	0.821	0.297
Provide transport to a place where your child can do PA or play sports	−0.11 (0.28)	0.703	0.406	2.41 (1.08)	**0.026**	0.460	2.32 (2.02)	0.251	0.742
Do a physical activity or play sports with your child	0.06 (0.29)	0.845	0.319	1.56 (1.14)	0.172	0.873	1.85 (2.08)	0.373	0.550

Data reported as beta coefficient and SE

*PA* physical activity, *MVPA* moderate- to vigorous-intensity physical activity, *SES* socio-economic status

^a^Associations between each independent variable and the dependent variable, adjusted for child age and sex, SES, and clustering by school. Values in bold indicate significance (*p* < 0.05)

## Discussion

It is important to understand the association between the neighborhood environment and the extent to which children accumulate MVPA outside of school hours to design targeted interventions to increase physical activity. The main objectives of this paper were to (1) assess whether parents’ perceptions of the neighborhood environment were associated with children’s out-of-school hours and weekend day MVPA, (2) assess whether objective measures of the neighborhood environment were associated with children’s out-of-school hours and weekend day MVPA, and (3) examine whether these associations differ between different income settings.

The majority of studies on neighborhood safety have focused on parental perceptions of safety rather than objective measures [4], and it has been argued that subjective ratings of the environment are a stronger predictor of behavior than objective measures [19]. For example, a study by Machado-Rodrigues et al. found that parental perceptions of neighborhood recreational facilities and infrastructure for walking and cycling was associated with habitual physical activity in seven to nine year old Portuguese girls [20]. In contrast, we found no association between any of the parental perceptions of neighborhood safety, traffic safety, walkability and social cohesion and children’s MVPA. This is consistent with more recent research from high income countries by Carson et al. [3] who also found no association between perceived neighborhood safety and children’s physical activity [3] and D'haese et al. [6] who did not find associations between perceptions of traffic safety, stranger danger, places to be physically active or sports venues and children’s MVPA [6].

In this study, we found a significant, positive association between the parents’ perception of the number of facilities available for physical activity in the neighborhood and before-school MVPA. This effect was moderated by SES, such that the positive association was only present in the low SES group. Our results showed that children in the high SES group had more access to motorized transport (76 %), while the majority of children in the low SES group (58 %) travelled to school using active transport, allowing the children in the low SES group the opportunity, or at least the perception thereof, to use physical activity facilities on their way to school [7]. It is also possible that parents are more likely to let their children use active transport to school when they perceive the neighborhood to have more facilities, as was seen in a study on 10 to 12 year old Belgian children who reported more active transport in girls when parents perceived higher recreational facilities and available walking/cycling infrastructure [10].

Objective measures of both neighborhood safety and traffic risk were negatively associated with after-school MVPA. That is, children engaged in less MVPA after school in areas with higher crime rates and greater traffic risk. This is similar to a recent study in Canada that found that objective measures of neighborhood safety and crime were independently associated with physical activity in free-time outside of school [16], and another study found significant inverse associations between objectively measured crime rates and outdoor physical activity in girls but not in boys [15]. We also found that SES had a significant moderating effect on these two objectively measured constructs (neighborhood safety and traffic risk). The low SES group participated in significantly less MVPA after school in neighborhoods that were unsafe and had a high traffic risk. Neighborhood safety and traffic risk were unrelated to MVPA in the high SES group. Children in low SES neighborhoods don’t always have the opportunity to be a member at a sports club, due to high costs [31] and are more likely to engage in less organized or more informal forms of physical activity, compared to children in high SES neighborhoods. For this reason, characteristics of the neighborhood environment are probably more important for enabling physical activity in low SES neighborhoods. These results indicate that although the perceived environment is important, the objective neighborhood environment seemed to be more strongly associated with children’s MVPA, at least in this setting. Furthermore, there were no associations between the objective neighborhood environment and weekend day MVPA. This is an interesting observation and could be attributed to the time periods of activity. On weekend days children have more freedom about when they choose to engage in physical activity, while during the week, they have specific after-school periods when they can be active in the neighborhood, and this could be periods where there is more traffic in the neighborhood as opposed to quieter weekends, indicating that after school time is the most critical time period for these types of pursuits.

In contrast to previous research [5, 23, 35], we found family support for physical activity (watching child do physical activity, providing encouragement and doing physical activity with child) to be unrelated to children’s out-of-school MVPA. We found a significant, positive association between providing transport to places for physical activity or sport and after-school MVPA, but not before school or on weekends.

A limitation of this study is that we can’t exclude the potential non-response bias as the analyses only included participants whose parents returned their questionnaires, reported their annual family income and provided a valid home address which could be geocoded. The sample slightly over-represented the low income group, particularly in higher income schools, and children’s height and MVPA in this sub-sample were significantly different from the group not represented. Furthermore, the study has a cross-sectional design, which limits inferences about cause-and-effect relationships. A strength of this study is the use of both perceived as well as objectively measured neighborhood constructs. The use of objectively measured physical activity as opposed to self-report physical activity further strengthens this study.

## Conclusion

This study found that a number of objective measures of the neighborhood environment were significantly associated with children’s outside-of-school MVPA, while most of the parents’ perceptions of the neighborhood environment had no effect. Furthermore, we found that SES plays a role - the perceived and objective measures of the neighborhood environment which were associated with MVPA, were significantly associated with the low-income group, but not with the high income group. Future interventions for the promotion of physical activity in children may need to focus more strongly on modifying these aspects of the neighborhood environment rather than trying to influence parent’s perceptions about their neighborhoods and greater attention should be given to low SES areas.

## Abbreviations

BMI: body mass index
GIS: Geographic Information Systems
ISCOLE: International Study of Childhood Obesity, Lifestyle and the Environment
MVPA: moderate- to vigorous-intensity physical activity
NIK: Neighborhood impact on kids study
PA: physical activity
SES: socio-economic status
WCED: Western Cape Education Department
